# Role of Alveolar β2-Adrenergic Receptors on Lung Fluid Clearance and Exercise Ventilation in Healthy Humans

**DOI:** 10.1371/journal.pone.0061877

**Published:** 2013-04-16

**Authors:** Stefania Paolillo, Riccardo Pellegrino, Elisabetta Salvioni, Mauro Contini, Annamaria Iorio, Francesca Bovis, Andrea Antonelli, Roberto Torchio, Carlo Gulotta, Alessandro Locatelli, Piergiuseppe Agostoni

**Affiliations:** 1 Centro Cardiologico Monzino, Istituto Di Ricovero e Cura a Carattere Scientifico, Milano, Italy; 2 Dipartimento di Medicina Interna, Scienze cardiovascolari ed immunologiche, Università Federico II, Napoli, Italy; 3 Allergologia e Fisiopatologia Respiratoria, ASO S. Croce e Carle, Cuneo, Italy; 4 Dipartimento cardiovascolare, Ospedali Riuniti e Università di Trieste, Trieste, Italy; 5 Pneumologia-Fisiopatologia Respiratoria, AOU S. Luigi, Orbassano, Torino, Italy; 6 Anestesia cardiochirurgica, ASO S. Croce e Carle, Cuneo, Italy; 7 Dipartimento di Scienze Cliniche e di Comunità, Università di Milano, Milan, Italy; 8 Division of Pulmonary and Critical Care Medicine, Department of Medicine, University of Washington, Seattle, Washington, United States of America; University Hospital Freiburg, Germany

## Abstract

**Background:**

In experimental conditions alveolar fluid clearance is controlled by alveolar β_2_-adrenergic receptors. We hypothesized that if this occurs in humans, then non-selective β-blockers should reduce the membrane diffusing capacity (D_M_), an index of lung interstitial fluid homeostasis. Moreover, we wondered whether this effect is potentiated by saline solution infusion, an intervention expected to cause interstitial lung edema. Since fluid retention within the lungs might trigger excessive ventilation during exercise, we also hypothesized that after the β_2_-blockade ventilation increased in excess to CO_2_ output and this was further enhanced by interstitial edema.

**Methods and Results:**

22 healthy males took part in the study. On day 1, spirometry, lung diffusion for carbon monoxide (DLCO) including its subcomponents D_M_ and capillary volume (V_Cap_), and cardiopulmonary exercise test were performed. On day 2, these tests were repeated after rapid 25 ml/kg saline infusion. Then, in random order 11 subjects were assigned to oral treatment with Carvedilol (CARV) and 11 to Bisoprolol (BISOPR). When heart rate fell at least by 10 beats·min^−1^, the tests were repeated before (day 3) and after saline infusion (day 4). CARV but not BISOPR, decreased D_M_ (−13±7%, p = 0.001) and increased V_Cap_ (+20±22%, p = 0.016) and VE/VCO_2_ slope (+12±8%, p<0.01). These changes further increased after saline: −18±13% for D_M_ (p<0.01), +44±28% for V_Cap_ (p<0.001), and +20±10% for VE/VCO_2_ slope (p<0.001).

**Conclusions:**

These findings support the hypothesis that in humans *in vivo* the β_2_-alveolar receptors contribute to control alveolar fluid clearance and that interstitial lung fluid may trigger exercise hyperventilation.

## Introduction

During acute fluid overload, gas exchange in the lungs is preserved as a result of at least two major mechanisms. First, part of the fluid is accumulated within the interstitial space and around the small airways, thus retarding the formation of alveolar edema [1], [2]. Second, fluid permeating the alveolar-capillary membrane is reabsorbed as a result of Na^+^ transport systems located on the alveolar surface [3], [4] and controlled by the β_2_ adrenoreceptor system [5], [6]. The involvement of these receptors has been reported in several animal and *ex-vivo* human lungs studies documenting an increase in alveolar fluid reabsorption upon stimulation of the β_2_-alveolar receptors by endogenous and exogenous catecholamine [4], [7], [8]. Fluid reabsorption from the alveolar and interstitial compartments to the vascular bed has also been invoked to explain the gradual recovery of gas exchange over time at high altitude [9], [10].

*In vivo* in humans fluid accumulation within the interstitial lung compartment can be estimated from the changes of lung diffusion capacity for carbon monoxide (DLCO) [11]. The test is the result of two in series resistive components with the first describing the passage of carbon monoxide (CO) through the alveolar-capillary membrane (membrane diffusion, D_M_), and the latter the combination of the gas with hemoglobin, from which the capillary volume (V_Cap_) can be computed [12]. In heart failure (HF), gradual accumulation of fluid across the lungs leads to a decrease of gas exchange capacity [13] presumably when fluid accumulation in the interstitial space and reabsorption by alveolar Na^+^ transport systems are fully exploited. Under these conditions, reduced DLCO and/or D_M_ have been consistently reported to be associated to a reduced exercise capacity [13], [14], reduced ventilatory efficiency [15] and poor prognosis [16]. β-blockers are among the cornerstone tools for HF treatment [17], [18] but substantial functional differences have been documented within this class of medications. For instance, in contrast to Bisoprolol (BISOPR), Carvedilol (CARV) has been shown to reduce DLCO in HF patients [19], [20]. This has been interpreted as a result of different mechanisms of these β-blockers on the alveolar β_2_-receptors and therefore on fluid flux across the lung, though this hypothesis has never been proved [21].

This study was conceived to clarify the role of the alveolar β_2_-receptors on reabsorption of lung fluid in humans *in vivo* and see whether interstitial lung edema affects the ventilatory efficiency during exercise. Our first postulate was that by blocking the β_2_-adrenoreceptors with CARV, a non-selective β_1_-β_2_blocker, D_M_ decreased as a result of the reduced lung fluid reabsorption. This hypothesis was tested in 11 healthy subjects by measuring the D_M_ changes after a short course of oral CARV. Eleven healthy subjects treated with BISOPR, a selective β_1_-blocker, represented the control group. To further explore the role of the alveolar β_2_-receptors in regulating fluid kinetics across the lung interstitium, the experiment was repeated after infusing saline solution, an intervention expected to increase lung fluid within the interstitial lung compartment [22]. Under these conditions we predicted that CARV would be associated with a further decrease in D_M_ as a result of the inability of the inactivated β_2_-receptors to clear the fluid out of the alveolar compartment. The second hypothesis that interstitial lung edema contributes to drive minute ventilation (VE) during exercise was tested in the same groups of subjects by examining the changes of the slope of the regression analysis between VE and carbon dioxide production (VCO_2_) as a function of D_M_ before and after CARV, BISOPR and saline solution infusion. The prediction was that if interstitial lung edema is a determinant of exercise hyperventilation, then the slope of VE *vs.* VCO_2_ should increase with the decrease in D_M_.

## Methods

### Patient population and study protocol

Twenty-two healthy volunteers selected from the medical staff participated in the study. Inclusion criteria were age between 20–60 years, male sex, no smoking history, absence of contraindications to β-blocker therapy, systolic blood pressure at rest ranging from 120 to 140 mmHg, diastolic blood pressure 70–80 mmHg, heart rate (HR) at rest >60 bpm, normal clinical evaluation, history, standard spirometry, echocardiogram and cardiopulmonary exercise test. Among the exclusion criteria were history and/or clinical evidence of any cardiovascular or pulmonary or systemic disorders contraindicating the test or affecting the functional response to exercise, any conditions requiring daily medications, and inability to adequately perform the required maneuvers for pulmonary function tests. The protocol was approved by the local Ethics Committee (Institutional Review Board no. S154/319, date13/04/2011), and written informed consent was signed prior to the study. The study was registered as EudraCT 2010-020357-14.

### Study protocol

#### Pre-study day

The subjects underwent clinical examination, including blood pressure, HR, and cardio-pulmonary exercise test (CPET) on a cyclo-ergometer for familiarization purposes. If the inclusion/exclusion criteria were met, the subjects were informed of the aim and protocol of the study and requested to sign the consent.

#### Study days

**Figure 1:**
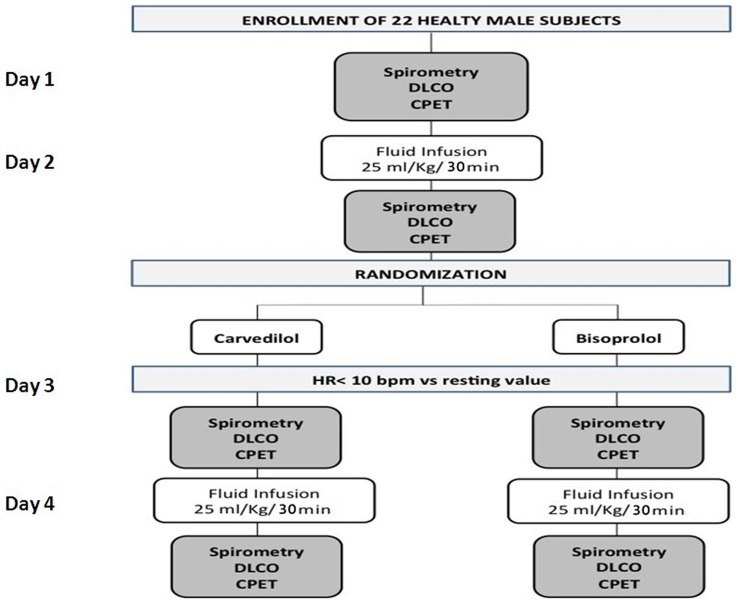
Design of the study. Legend: DLCO = Lung diffusion for carbon monoxide; CPET = Cardiopulmonary exercise test; HR = Heart rate.

The subjects attended the laboratory on four different occasions whose sequence is shown in Figure 1. On day 1, standard pulmonary function tests, DLCO measurement including its subcomponents D_M_ and V_Cap_, and CPET were performed. On day 2, the same tests were repeated after infusion of saline solution at the dose of 25 ml/Kg in less than 30 min. Then, the subjects were randomized to receive either oral CARV (11 subjects) or BISOPR (11 subjects), respectively. In the following days the dose of β-blocker was up-titrated until HR decreased by at least 10 beats·min^−1^. Thereafter, lung function tests, DLCO, D_M_, V_Cap_ and CPET were retested before (day 3) and after saline solution infusion (day 4). β-blocker treatment was conducted for at least 5 days to achieve the desired decrease in HR. Study days 1 and 2 and study days 3 and 4 were separated by no more than 48 hours from each other.

### Spirometry and DLCO measurements

Spirometry was performed according to current guidelines with a mass flow-meter (SensorMedics, Yorba Linda, CA) [23]. Predicted values are from Quanjer et al. [24].

DLCO and its subcomponents D_M_ and V_Cap_ were measured with the single-breath method by breathing gas mixtures containing three different O_2_ concentrations (21%, 40% and 60%) and 0.3% CO and 0.3% methane (CH_4_) according to Roughton and Forster [12]. DLCO after saline infusion was corrected for a difference of Hb of 0.57 g/dL *vs.* control conditions, a value derived from a previous study conducted with the same amount of saline/Kg [25]. Alveolar volume was estimated from the CH_4_ decay slope during constant expiratory flow measurement [26]. Measurements at each mixture were performed at least in duplicate and according to the standard requirements and acceptability criteria of current guidelines [11]. The gas exchange measurements were performed with a V-max 2900 metabolic cart (Sensor Medics, Yorba Linda, CA).

### Cardiopulmonary exercise test

A symptom-limited incremental exercise test was performed on an electronically braked cycle ergometer (Erg 800S, SensorMedics, Yorba Linda, CA). A personalized ramp protocol was designed to achieve maximum load in 10±2 minutes in each subject [27]. The volunteers wore a nose clip and breathed through a mass flow sensor connected to a saliva trap. Ventilation and respiratory gases were measured breath by breath (V-max 2900 metabolic cart, Sensor Medics, Yorba Linda, CA) and then averaged every 20 s for analysis. HR and 12-lead ECG were monitored continuously, hemoglobin saturation was recorded by an ear oxymeter, and blood pressure was monitored with a cuff sphygmomanometer at rest and at peak exercise. Anaerobic threshold (AT) was measured according to standard technique [28]. VE/VCO_2_ slope was measured from the beginning of loaded pedaling to the end of the isocapnic buffering period [29].

### Statistical analysis

Data are reported as mean ± SD. Differences between study days or treatment groups were analyzed as percent changes of day 1. The effects of saline infusion were examined by paired Student's *t*-test, whereas the effects of medications with and without saline infusion by unpaired Student's *t*-test. Statistical significance was accepted at p<0.05 to reject the null hypothesis. Correlation between variables was assessed by linear regression analysis. The sample size of 11 subjects per group provided a 90% power to detect changes in D_M_ by 10 mL·mmHg^−1^·min^−1^ and VE/VCO2 slope by 5 L·min^−1^ after CARV and saline infusion with an alpha = 0.05.

Data were stored in an Excel database and then analyzed by SPSS 17.0 (SPSS Inc., Chicago, IL)..

## Results

The main anthropometric and functional data at rest and peak exercise of day 1 are reported in Table 1.

**Table 1:** Main anthropometric and functional parameters at rest and peak exercise.

Number of subjects	22
Age, years	40±12
Height, cm	180±10
BMI, kg·m^−2^	25.3±3.6
FEV_1_, L (% pred)	4.20±0.60 (107±11)
FVC, L (% pred)	5.10±0.60 (105±11)
DLCO, mL·mmHg^−1^·min^−1^ (% pred)	31.8±4.7 (97±12)
D_M_, mL·mmHg^−1^·min^−1^	57.2±12.3
V_Cap_, mL	94.4±25.5
VA, L	6.4±0.7
Load, watt (% pred)	206±35 (95±18)
HR max, min^−1^ (% pred)	167±13 (96±5)
VO_2_ peak, ml·min^−1^ (%)	33.5±6.4 (90±17)
VE/VCO_2_ slope	23.1±3.1

Legend: BMI = Body mass index; FEV_1_ = Forced expiratory volume in 1 second; FVC = Forced vital capacity; DLCO = Diffusing lung capacity for carbon monoxide; D_M_ = Membrane diffusion; V_Cap_ = Capillary volume; VA = Alveolar volume; HR = Heart rate; VO_2_peak = Oxygen uptake at peak exercise; VE/VCO_2_ slope  = slope of the linear regression analysis of VE plotted *vs.* VCO_2_.

### Effects of saline infusion (day 2)

**Figure 2:**
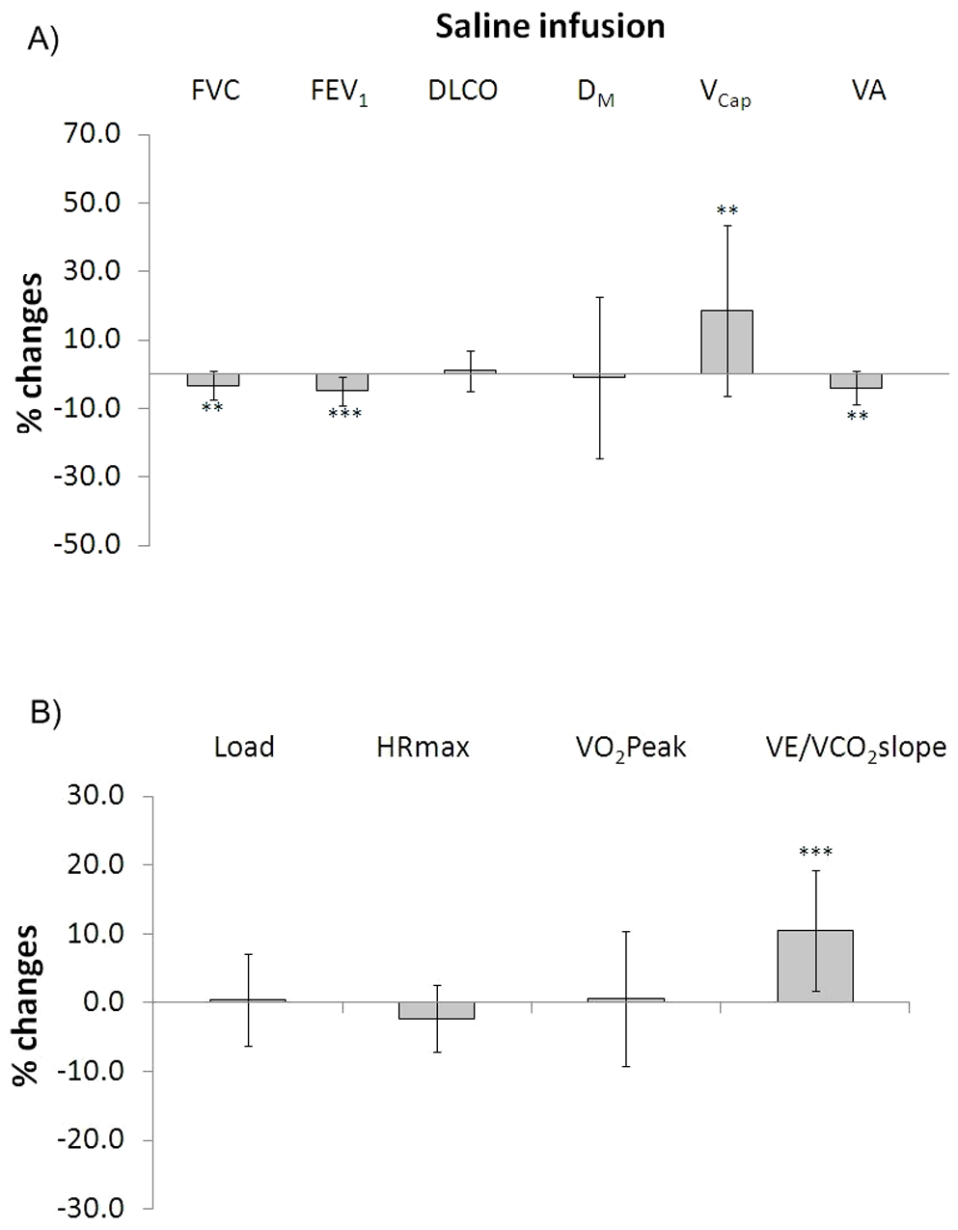
Changes of the main functional parameters at rest (panel A) and with exercise (panel B) after saline infusion with respect to baseline conditions. Legend: FVC = Forced vital capacity; FEV_1_ = Forced expiratory volume in 1 second; DLCO = Lung diffusion for carbon monoxide; D_M_ = Membrane diffusion; V_Cap_ = Capillary volume; VA = Alveolar volume; HR = Heart rate; VO_2_peak = Oxygen uptake at peak exercise; VE/VCO_2_slope = slope of the linear regression analysis of VE plotted *vs.* VCO_2_ from the beginning of loaded pedaling to the end of the isocapnic buffering period. Statistical differences were examined by paired Student's *t*-test. Symbols denote statistical significance (** = p<0.01; *** = p<0.001).

Saline solution infusion was associated with a slight but significant decrease in FVC, FEV_1_ and alveolar volume. D_M_ and DLCO remained unchanged whereas V_Cap_ increased (p = 0.006). Exercise capacity was not significantly modified by the intervention. Ventilation efficiency was significantly reduced after saline infusion as documented by the 10±9% increase of the VE/VCO_2_ slope (p<0.001). No correlations were found between the changes in VE/VCO_2_ and any of the DLCO parameters after saline infusion. The main results are shown in figures 2A and B.

### Effects of β-blockade (day 3)

**Figure 3:**
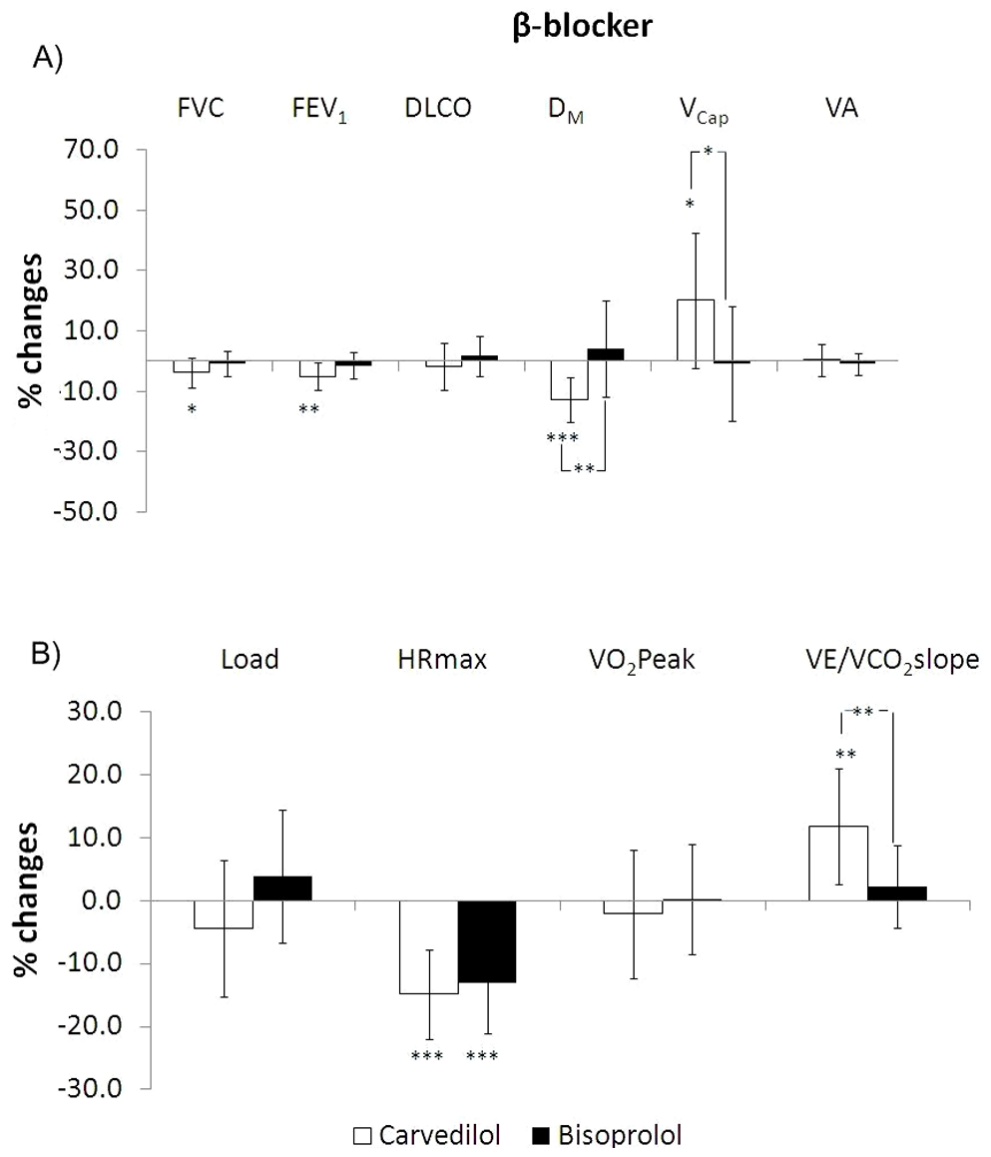
Changes in the main functional parameters at rest (panel A) and with exercise (panel B) after Carvedilol (empty figures) and Bisoprolol (full figures) with respect to baseline conditions. Statistical differences were examined by unpaired Student's *t*-test. Legend as in figure 2. Symbols denote statistical significance (* = p<0.05; ** = p<0.01; *** = p<0.001).

Average daily doses of CARV and BISOPR were 25±0 mg and 6.6±2.8 mg, respectively. Both treatments led to similar decrements in resting HR (17±7 and 15±7 beats·min^−1^ for CARV and BISOPR, respectively). FEV_1_ and FVC slightly but significantly decreased with CARV (p<0.05 and p<0.01, respectively) but not with BISOPR. In contrast, D_M_ and V_Cap_ exhibited significantly larger changes with CARV (p<0.01 and p<0.05, respectively) than BISOPR. Again, DLCO remained unmodified by any treatments. At peak exercise HR was lower than at day 1 in both groups (p<0.001 for both). The VE/VCO_2_ slope significantly increased with CARV compared to BISOPR (p<0.001). None of the other parameters were significantly modified by the β-blockade. No correlations were observed between the changes in VE/VCO_2_ and in DLCO or any of its subcomponents after either β-blocker treatments. The main results are shown in figures 3A and 3B.

### Effects of saline solution infusion under CARV and BISOPR conditions (day 4)

**Figure 4:**
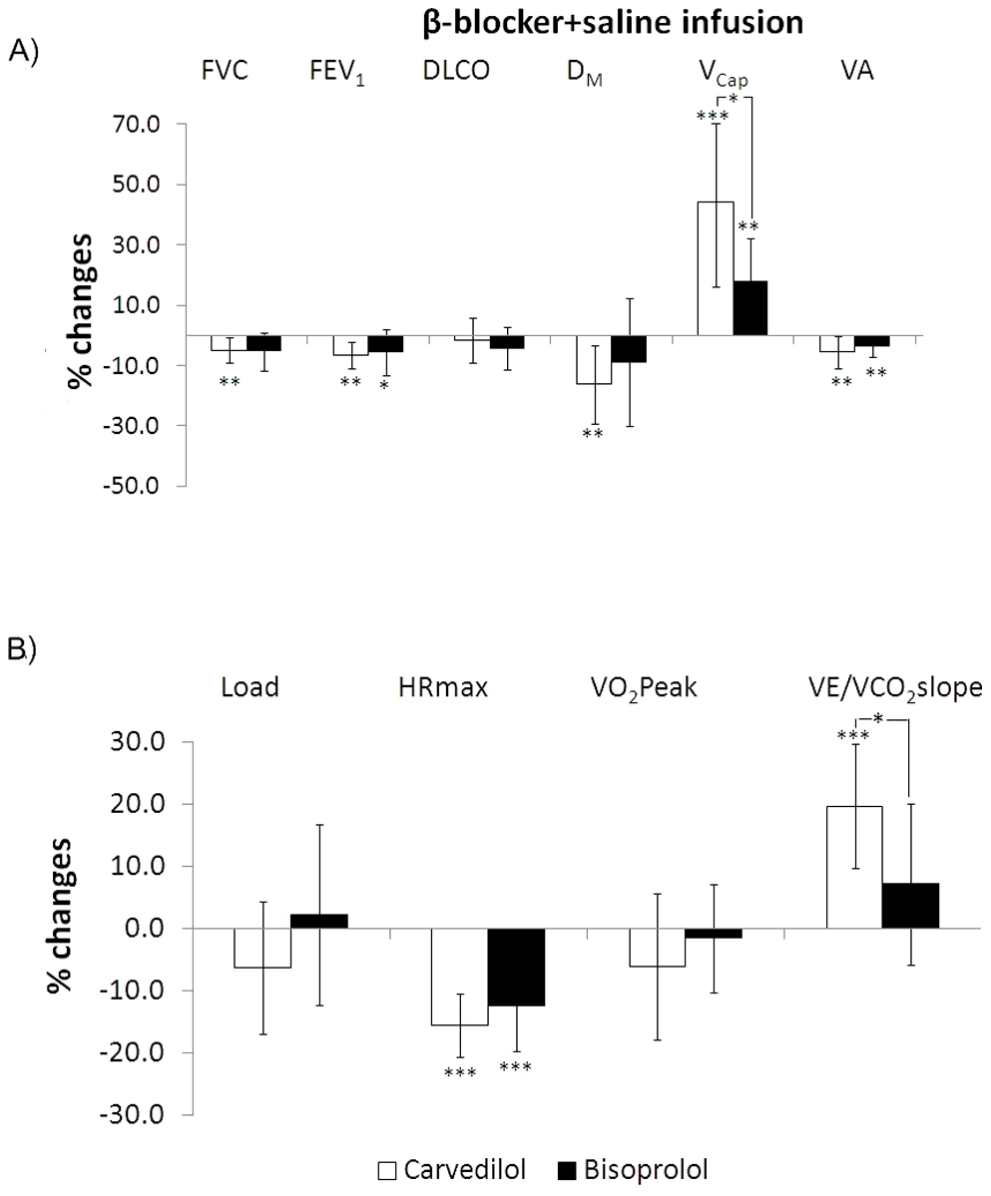
Changes in the main functional parameters at rest (panel A) and with exercise (panel B) after saline infusion under Carvedilol (empty figures) and Bisoprolol (full figures) conditions with respect to baseline conditions. Statistical differences were examined by unpaired Student's *t*-test. Legend as in figure 2. Symbols denote statistical significance (* = p<0.05; ** = <0.01;*** = p<0.001).

After either β-blockers, saline infusion caused mild reductions in FEV_1_ and FVC. With CARV D_M_ and V_Cap_ were significantly reduced and increased, respectively. Their changes were larger than at day 3 (p<0.02 for both). With CARV the increase in V_Cap_ was larger than with BISOPR (p<0.02) (figure 4A). Exercise capacity was preserved with either treatments. However, the VE/VCO_2_ slope significantly increased more with CARV than BISOPR (figure 4B) (p = 0.02). No significant correlations were found between the changes in VE/VCO_2_ and in any lung diffusion parameters with CARV.

## Discussion

The main results of this study are that, compared with BISOPR, CARV was associated with a significant reduction in D_M_ and increment in V_Cap_, that were larger when the test was preceded by saline infusion. As CARV is a non-selective β-blocker, these findings support the hypothesis that alveolar β_2_-adrenergic receptors play a significant role in controlling alveolar fluids reabsorption *in vivo* in humans. In addition, the significant increment in VE/VCO_2_ observed with CARV but not with BISOPR either before or after saline solution infusion suggests that interstitial lung edema may contribute to exaggerate the ventilatory response to exercise in humans.

### Interpretation of results and limits of the study

β_2_-adrenergic receptors are expressed all throughout the lung including the alveolar space where they regulate several key proteins needed for ion and fluid transport. This has been documented in rat and sheep alveolar epithelial cells as well as in human lung cells [30]. Even though also the alveolar β_1_-adrenergic receptors can accelerate alveolar active sodium transport [31], data on alveolar β_2_-adrenergic receptors knockout mice suggest that it is the β_2_ receptors that are responsible for the most of the β-receptor mediated fluid reabsorption [32]. No data are available *in vivo* in humans.

To test the hypothesis that alveolar β_2_-adrenergic receptors contribute to regulate fluid homeostasis within the alveolar compartment in humans *in vivo* we examined the changes in D_M_, a very sensitive marker of fluid accumulation within the interstitial lung compartment, after blocking the alveolar adrenergic receptors and over-hydrating the lung. With the latter intervention, V_Cap_ increased presumably as a result of the recruitment or over-distension of the pulmonary capillaries. This was associated with a decrease in D_M_ consistent with fluid accumulating also along the alveolar capillary membrane and presumably within the space around the small airways. DLCO did not change as a result of the opposite changes in D_M_ and V_Cap_ that compensated each other. A very mild restrictive pattern also occurred, as suggested by the decrease in FEV_1_ and FVC. These data are in line with previous reports, where infusion of a similar amount of saline had similar effects in lung function [33], [34], [35], [36] and DLCO [6], [25], [37].

With CARV, D_M_ significantly decreased, presumably because the β_2_-blockade was such that the active pumps located on the alveolar surface and necessary to pump fluid out of the alveolar compartment were down-regulated. However, the increase in V_Cap_ was quite a surprising finding and difficult to explain. Because β-blockers are not known to increase cardiac output in healthy subjects, we postulate that the increase in V_Cap_ could have been the result of some local feed-back control to maintain constant gas exchange counterbalancing D_M_ reduction [13]. Can these data support the hypothesis that the alveolar β_2_-receptors *in vivo* in humans contribute to keep fluid homeostasis across the alveolar-interstitial membrane? We believe this is the case for the following reasons. First, D_M_ is a very sensitive functional index of the alveolar-capillary membrane as it estimates the CO gradient between the two compartments [12], and as such, is a reflection of the interstitium morphology [13]. As there were no other reasons for D_M_ to decrease in our study, these findings strongly suggest that blocking the alveolar β_2_-adrenergic receptors with CARV allowed some fluid to be accumulated within the lung interstitium. Second, in animals models, infusion of saline solution was associated with interstitial and airway wall edema [1], and in HF infusion of even small amount of saline was associated with gas exchange worsening [37], [38]. What our study documents is that pharmacological inhibition of the β_2_-receptors led to functional findings very similar to saline infusion at the level of the alveolar-capillary membrane. Given the bulk of evidence that alveolar β_2_-receptors regulate fluid clearance through the active Na^+^ channels at least in vitro [3], [5], the decrease in D_M_ after CARV lends support for the first time to the hypothesis that the alveolar β_2_-receptors control the fluid homeostasis *in vivo* in humans across the alveolar-capillary membrane.

**Figure 5:**
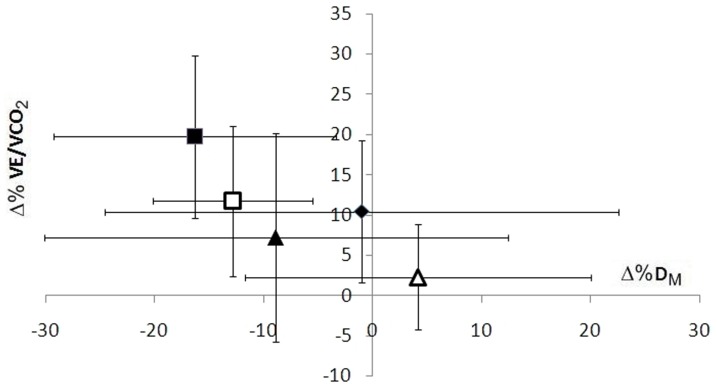
Mean changes in VE/VCO_2_ slope *vs.* D_M_ caused by saline infusion alone (diamond), Bisoprolol (empty triangle), Carvedilol (empty square) and saline infusion with Bisoprolol (full triangle) and Carvedilol (full square).

In animals, induced pulmonary vascular congestion stimulates the pulmonary C-fibers [39], [40] that in turn trigger rapid shallow breathing [41]. Studies in HF patients [15] and in healthy subjects before and after saline infusion documented an excess of ventilation with respect to the CO_2_ produced during exercise [25], suggesting that interstitial lung edema could contribute to cause hyperventilation during exercise. At a first glance, our findings appear to negate this hypothesis as no correlations were observed between the increase in VE/VCO_2_ slope and decrease in D_M_. Yet despite the lack of this kind of evidence we believe that the idea that interstitial lung edema can trigger exercise hyperventilation is still true for a series of reasons. First, the increase of the VE/VCO_2_ slope was always coincident with the decrease of D_M_ no matter how the latter was achieved with saline solution infusion, CARV, or both. When BISOPR was given instead of CARV, D_M_ remained unmodified by treatment, and so did the VE/VCO_2_ slope. Second, when the changes in VE/VCO_2_ were plotted against the changes in D_M_ on all study days, a clear relationship became apparent (figure 5), suggesting that VE increased in excess to the VCO_2_ when D_M_ was reduced. If this reinforces our original hypothesis that interstitial lung edema significantly contributes to reduce the ventilator efficiency during physical exercise, this does not deny that other mechanisms regulate ventilation during exercise especially in disease. For instance, in chronic HF, CARV, as opposed to BISOPR and the present findings in healthy humans, has been shown to reduce the VE/VCO_2_ slope [39] presumably because of its effects on the overactivated chemoreflex. Therefore, we acknowledge the overly simplicity of our model with respect to HF, a disorder where much of exercise hyperventilation is sustained not only by interstitial lung edema but also altered autonomic reflex control, lung vascular pathology and functional dysfunction, physical deconditioning, early occurrence of metabolic acidosis with exercise, and neural afferent signals from exercising muscles and systemic circulation [42], [43], [44]. As a second limit of the study, we also concede that a random cross-over design of the study could have provided more solid evidence about the effects of CARV on the alveolar β_2_-receptors blockade with respect to BISOPR. Yet, in designing the trial we considered this at a higher risk of drop-out than the double-blind random design because of the excessive burden of invasive tests and treatment for the participants.

### Clinical implications

HF is a complex disease where fluid accumulation within the interstitial space and exercise hyperventilation are the only two features reproduced in our model. Yet, our findings may offer important considerations for the clinical approach to the disease.

If interstitial lung edema is a key issue of the disease and presumably plays a substantial role to reduce the ventilatory efficiency during exercise, then it is wondered what is the best functional index for clinical follow-up. The decrease of FEV_1_ and FVC with CARV was significant from a statistical point of view but quite small and on average well within the limits of natural variability of the measurements over time [23], thus limiting their use in routine practice. DLCO appears to be a quite insensitive parameter to examine the perturbations of gas exchange with β-blockers and fluid accumulation within the lung interstitium. This is because the changes in its subcomponents tend to compensate each other, thus leaving DLCO unchanged. Measuring D_M_ appears to be a realistic alternative as it directly estimates the morphological changes at the alveolar-capillary membrane level. It remains to demonstrate the impact of such a functional test in clinical practice.

As a corollary, our data also suggest that non-selective β-blockers are not indicated in the presence of clinical and/or radiological signs of fluid accumulation within the lung.

### Conclusions

The present study shows that in healthy subjects CARV, a non-selective β_1_ and β_2_ blocker, was significantly associated with a decrease in D_M_, especially after saline infusion. As this finding was not replicated with BISOPR, a selective β_1_ blocker, we speculate that the decrease in D_M_ was the result of a blockade of the alveolar β_2_-adrenergic receptors known to control the active Na^+^ transport of fluid out the alveolar and interstitial lung compartments. The decrease in D_M_ was associated with an increase in the VE *vs.* VCO_2_ slope during exercise, thus suggesting that fluid accumulation within the interstitial lung compartment may contribute to trigger hyperventilation during exercise, thus reducing the ventilator efficiency. These findings may represent the basis for a more physiologically oriented β-blocker use in HF.
